# Improving metabolic flux predictions using absolute gene expression data

**DOI:** 10.1186/1752-0509-6-73

**Published:** 2012-06-19

**Authors:** Dave Lee, Kieran Smallbone, Warwick B Dunn, Ettore Murabito, Catherine L Winder, Douglas B Kell, Pedro Mendes, Neil Swainston

**Affiliations:** 1 Manchester Institute of Biotechnology, University of Manchester, 131 Princess Street, Manchester, M1 7DN, UK; 2 School of Chemistry, University of Manchester, Manchester, M13 9PL, UK; 3 Virginia Bioinformatics Institute, Virginia Tech, Washington St. 0477, Blacksburg, Virginia, 24060, USA

**Keywords:** Flux balance analysis, Metabolic flux, Metabolic networks, Transcriptomics, RNA-Seq, Exometabolomics

## Abstract

**Background:**

Constraint-based analysis of genome-scale metabolic models typically relies upon maximisation of a cellular objective function such as the rate or efficiency of biomass production. Whilst this assumption may be valid in the case of microorganisms growing under certain conditions, it is likely invalid in general, and especially for multicellular organisms, where cellular objectives differ greatly both between and within cell types. Moreover, for the purposes of biotechnological applications, it is normally the flux to a specific metabolite or product that is of interest rather than the rate of production of biomass *per se*.

**Results:**

An alternative objective function is presented, that is based upon maximising the correlation between experimentally measured absolute gene expression data and predicted internal reaction fluxes. Using quantitative transcriptomics data acquired from *Saccharomyces cerevisiae* cultures under two growth conditions, the method outperforms traditional approaches for predicting experimentally measured exometabolic flux that are reliant upon maximisation of the rate of biomass production.

**Conclusion:**

Due to its improved prediction of experimentally measured metabolic fluxes, and of its lack of a requirement for knowledge of the biomass composition of the organism under the conditions of interest, the approach is likely to be of rather general utility. The method has been shown to predict fluxes reliably in single cellular systems. Subsequent work will investigate the method’s ability to generate condition- and tissue-specific flux predictions in multicellular organisms.

## Background

The applications of genome-scale metabolic modelling have increased over recent years, as have the number of metabolic models available and the diversity of organisms that such reconstructions cover [1]. Traditional approaches to analysing such models have focused on constraint-based modelling, including widely used techniques such as flux balance analysis (FBA) [2]. FBA relies upon specification of an objective function that the cell is assumed to optimise. Objective functions can cover a range of cellular objectives [3], such as maximisation / minimisation of ATP consumption, but frequently (and particularly in the case of microorganisms) take the form of an assumed “biomass” function; a hypothetical reaction that mimics cell growth rate [4]. Such a biomass function is used to account for the flow of materials that are necessary for building new cells, and is commonly required in constraint-based models even when maximising variables other than growth rate.

Maximisation of biomass yield is not generally considered a valid principle in microbiology [5]; however, it is commonly assumed to be the optimality criterion driving the evolution of microorganisms [6]. The premise is that an organism that acquires and/or redistributes resources to outgrow its competitors will be in the best position to survive [7,8]. Assumption of growth rate maximisation in FBA studies has led to successful predictions of the actual growth rate in a number of organisms [4,9]. Such an assumption, however, is likely to be invalid for individual cell types in multicellular organisms, where cellular objectives may differ greatly both between and within tissues. The assumption of maximal rates of biomass production involves an objective at the cellular level, whereas in multicellular organisms a given cell’s objective is likely to be realised via survival at the organism level, which may not necessarily be dependent upon the growth of the cell. Moreover, signals from the extracellular environment may trigger different cellular priorities and objectives. These issues have been demonstrated by the work of Gille *et al*. [7], who modelled the metabolism of human hepatocyte, indicating their objective is to preserve homeostasis of blood compounds, a process that is modulated by the extracellular availability of oxygen and other nutrients.

Another concern when applying FBA is the inadequacy of the biomass definition itself. Typically, the assumed composition of *E. coli* is used as a template to define the biomass of the organism of interest [4]. To do this, a cell’s macromolecular composition (in terms of protein, RNA, DNA, carbohydrate and lipid content), the metabolite content of each macromolecular class, and the biosynthetic and maintenance costs for various cellular processes are required [4]. Not only are such numbers difficult to determine, but it is also to be expected that they would change drastically under different environmental conditions. Problems associated with reliance on a biomass objective function have led to a number of studies that focus on the determination of a suitable objective function [10-12].

This work therefore focuses on another approach, investigating the use of ‘omics data to act as a guide for the prediction of the intracellular metabolic fluxes that a given cell exhibits. *A priori* it may be supposed that enzymatic transcript concentrations and metabolic fluxes can be related to each other, albeit in a complex manner, since fluxes are clearly dependent on the concentrations of enzymes and/or their encoding transcripts [13]. Drawing upon previous work [14-17], this approach investigates how relating metabolic fluxes to enzyme-encoding gene expression levels affects the predictive power of constraint-based analysis. The hypothesis is that doing so would provide a comparable, or better, representation of intracellular fluxes than does reliance upon an assumed biomass objective alone. As stated in related work by Becker *et al*. [18], “the statement of an [assumed] objective introduces a ‘user-bias’ and such objective may not be relevant to the true physiological state.” It is the removal of this user-bias, through the application of a purely data-driven objective, that this work attempts to address.

This study involves the acquisition and analysis of absolute quantitative transcriptomics data from *Saccharomyces cerevisiae*, and the subsequent use of these data to constrain an existing genome-scale metabolic model [19]. A comparison between FBA results generated through this approach and those generated from applying a more traditional biomass objective is performed.

Such an approach may have ramifications for the genome-scale modelling of human metabolism [20], where tissue-specific microarray data are publicly available and have been exploited in the development of tissue-specific models of human metabolism by Shlomi *et al*. [14]. The approach of Shlomi *et al*. categorises numerous tissue-specific relative gene expression data sets into high, medium and low expression, with the goal of limiting the tissue-specific network to contain only those reactions for which consistent high gene expression has been observed. A disadvantage of this work is that it was performed upon a “consensus” of gene expression data, generated under a range of physiological and experimental conditions. Furthermore, the data used are generated from microarrays, which provide *relative* expression ratios of the same gene under different conditions. Microarrays are applicable to comparative studies, and as such, the data that they produce do not allow for comparison of absolute expression levels *across* genes, primarily due to differences in hybridisation efficiency [21]. In addition, microarray data is associated with a number of common problems, including cross-hybridisation issues, limited dynamic detection range, presence of background noise and the detection of transcripts being limited to sequences printed on the array [22].

The approach provided here relies upon *absolute* gene expression data generated under the condition of interest, using RNA-Seq. RNA-Seq provides expression levels in terms of counts of expressed transcripts that can be related to transcripts per cell and thus an absolute level. Therefore, the expression levels generated are comparable across the transcriptome and have been shown to be more indicative of protein concentrations than gene expression levels generated from microarrays [21,23]. By extension, RNA-Seq data is likely to provide a more reliable indication of enzymatic activity than that generated through relative expression techniques. Furthermore, RNA-Seq mitigates many of the limitations inherent in the use of microarrays [24].

## Results and discussion

**Figure 1:**
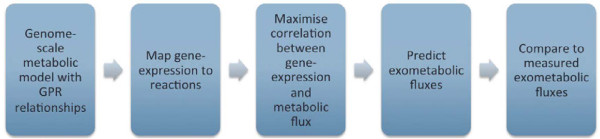
**Steps undertaken in constraining metabolic models with gene expression data.** The approach is applicable to genome-scale metabolic model that contain gene-protein-reaction (GPR) relationships. Absolute gene-expression data is mapped to individual reactions following the Boolean logic described in the “Mapping gene expression data to metabolic reactions” section of the Methods. Correlation between this gene-expression data and metabolic fluxes is maximised by following a three step algorithm comprising of: i) maximising the correlation between the initial set of irreversible reactions and the experimental data; ii) performing flux variability to determine additional reactions that must now be unidirectional; iii) repeating this cycle of maximising correlation until no extra irreversible reactions are found through flux variability analysis. The solution predicts exometabolic fluxes that can then be compared to those generated experimentally.

The methodology involves a number of steps. Specifically, these are i) providing gene-protein-reaction (GPR) relationships in the metabolic model; ii) mapping of gene expression data to individual reactions; iii) correlating gene expression data to the predicted metabolic flux; and iv) validating the metabolic flux predictions through comparison of predicted values against those determined experimentally. It is important to note that the model is not constrained with experimentally measured flux parameters. These values are subsequently used to validate the flux predictions generated solely from gene expression data. The steps involved in the method are described in detail below and summarised in Figure 1.

### Mapping gene expression data to metabolic reactions

Previous approaches to applying gene-level data to metabolic maps have involved thresholding; the gene can be defined as having two states: on or off [15], or three states: low, medium or high expression [14]. Here a different approach is used, where the absolute gene expression data are used to create continuous, rather than discrete, reaction weightings.

The method is applied to the genome-scale model of yeast metabolism, Yeast 5 [19], an extension of earlier community-driven yeast metabolic reconstructions [24,25]. RNA-Seq data are used to constrain the reconstruction, providing an absolute measurement of gene expression that is comparable across all genes in the data set. The hypothesis is that absolute and comparable measurements of gene expression can be used to infer activity of the encoded enzymes. Although it is recognised that there is overall poor correlation between gene and protein expression [26], recent studies have indicated an increased correlation between mRNA and protein concentrations than was reported previously [23,27]. The assumption behind the method is that gene expression levels can be used to infer metabolic fluxes with reasonable accuracy, provided that they are applied on a genome-scale, and provided that the predicted metabolic flux pattern adheres to the normal stoichiometric and thermodynamic constraints that are applied to constraint-based modelling. By applying gene expression levels on such a scale, and by adhering to such constraints, it is hoped that effects such as experimental error, differing protein translation rates, differing transcript and protein stabilities, and differing enzymatic activities, will be reduced. Whilst the procedure is here applied to transcriptomics data, future work will investigate whether the method would be improved by integration with absolute proteomics data [28].

The assignment of gene expression data to individual metabolic reactions is complicated by the fact that there is not a one-to-one mapping between gene and reaction. The presence of promiscuous enzymes [29], isoenzymes and enzymatic complexes underscores the requirement for a careful mapping of gene expression data to metabolic reactions. Details of the approach undertaken here are provided in the Methods section.

### From metabolic reactions to flux predictions

Constraint-based analysis [30,31] uses physicochemical constraints such as mass and charge balance, energy balance, and flux limitations to describe the potential behaviour of an organism.

The basic biochemical structure of the metabolic pathways is generally of sufficient granularity to allow deduction of the underlying network stoichiometry. In addition, the flux of each reaction through the system may be constrained through, for example, knowledge of enzyme maximal activities (*v*_*max*_) or irreversibility considerations [32]. Given a typical genome-scale metabolic model such as Yeast 5, at steady state these constraints may be written as:

(1) $$\begin{array}{c} Nv=0\\ L_{i}\leq v_{i}\leq U_{i} \end{array}$$

where *N* is the stoichiometric matrix, *v* a steady state flux pattern, and *L*_*i*_ and *U*_*i*_ the lower and upper flux bounds respectively for each reaction *i*.

A number of techniques have been proposed to deduce the network behaviour from this minimal set of information, including FBA and extreme pathway [33] or elementary mode analysis [34]. In particular, FBA highlights the most effective and efficient paths through the network in order to optimise a particular objective function [35]. Genome-scale reconstructions typically contain a pseudoreaction representing growth (a “biomass reaction”) and FBA is typically used to identify a pattern of fluxes at steady state that maximise the rate of this reaction. This is seen as corresponding to maximising the rate of biomass production with respect to a nutrient of reference (usually glucose), with other necessary nutrients assumed to be in excess. This is unlikely to be applicable to turbidostat cultures where growth rates are maximised but where their efficiencies in terms of mass of cells per mass of glucose may be low.

In this study, the rate of biomass production is not the function that is maximised explicitly. Instead, the focus is on maximising the correlation between the steady-state pattern of the predicted fluxes and the corresponding gene expression data at the biochemical level. This is achieved through minimising:

(2) $$Z=\sum_{i}\frac{1}{\sigma_{i}}\left|v_{i}-d_{i}\right|$$

where the sum is taken over all reactions *i* for which reaction data *d*_*i*_ are available, in this case, all reactions with an associated gene. *σ*_*i*_ is the error in data point *i* as calculated in the mapping of gene expression data to individual reactions (see Methods). Thus the objective is to predict a flux that is as close as possible to the reaction estimate, weighted according to the confidence in that estimate. The function *Z* is convex in *v*, and this allows the use of the powerful tools of convex programming [36]. Convexity is normally required to solve optimisation problems involving thousands of variables and is certainly to be preferred from the point of view of computational performance. This is the reason that a possibly more natural function such as the Pearson product-moment correlation coefficient is not used in its place. Moreover, *Z* may be linearised through decomposing *v* into components greater than, or less than, *d*:

(3) $$\begin{array}{l} Z=\sum_{i}\frac{1}{\sigma_{i}}\left(d_{i}^{+}+d_{i}^{-}\right)\\ v_{i}=d_{i}+d_{i}^{+}-d_{i}^{-}\\ d_{i}^{+},d_{i}^{-}\geq 0 \end{array}$$

for each *i*. Hence the fast and robust tools of linear programming [37] may be utilised.

One remaining problem is that the direction of reversible reactions (defined operationally as those whose net fluxes may be in either direction in the organism of interest) is potentially arbitrary. The aim is to maximise correlation between the absolute flux and reaction data, but replacing *v* with |*v*| in the definition of *Z* would remove its convexity. Instead, an iterative process is used to assign the data to the model:

1. Maximise the correlation between the initial set of irreversible reactions and the experimental data. The sum in *Z* is taken only over irreversible reactions (i.e. those with a known direction of flux).

2. Perform flux variability analysis (FVA) [38] to determine which further reactions in the network must thus be unidirectional, i.e. they cannot carry a flux in the other direction without decreasing the correlation coefficient found in point 1.

3. A new larger set of irreversible reactions is defined. The cycle between FVA and maximising correlation is continued until no extra irreversible reactions are found through variability analysis. As the number of irreversible reactions will never decrease, the algorithm terminates in a finite number of steps.

### Comparison of predicted and measured exometabolome fluxes

Fluxes were predicted by following two approaches: standard FBA (i.e. maximisation of cellular biomass), and maximisation of the correlation between specific metabolic fluxes and gene expression data, as described here. In the latter case, gene expression data were collected under two growth conditions: at 75% and 85% of the maximum biomass levels attainable for this strain with the utilised growth medium. In general FBA problems are underdetermined, i.e. there is an infinite number of solutions satisfying the criterion of optimality. The innumerable set of these equally optimal solutions is known as the solution space and is in the shape of a multi-dimensional polytope. Different algorithms have been proposed to explore the solution space of an FBA problem and enumerate its edges (the alternate optima) [33,39]. The approach followed here is to identify one single solution for each FBA formulation that is representative of the entire solution space. This is achieved by applying geometric FBA, a method that identifies the FBA solution that lies in the centre of the solution space [40].

The exometabolome or “metabolic footprint” of an organism provides a quick and convenient summary of its metabolic activities, especially under unbalanced growth conditions [41,42]. The predicted exometabolome fluxes from both methods were compared against experimentally measured fluxes. The coefficient of determination *R*^2^ was used to test the goodness of fit of the predictions; *R*^2^ = 1 means a perfect match between model and data, whilst *R*^2^ = 0 is the value obtained by simply predicting the mean of the data for each point. Thus *R*^2^ < 0 denotes a very poor prediction.

The results for the flux to exit of six metabolites - carbon dioxide, ethanol, glycerol, acetate, lactate and trehalose - are presented in Tables 1 and 2. These exchange fluxes were normalised according to the measured glucose uptake flux of their respective growth condition. The standard FBA method of maximising growth predicts that all glucose is converted to carbon dioxide, and provides a very poor fit to the data (*R*^2^ < 0). The best fit possible under the assumption of maximal biomass prediction is *R*^2^ = 0.20 and *R*^2^ = 0.58, respectively. In contrast, a good fit is found by applying the method described here to gene expression data gathered at both the 75% and 85% biomass level *R*^2^ = 0.87 and *R*^2^ = 0.96, respectively).

**Table 1:** Comparison of experimental with predicted exometabolome fluxes, at 75% maximal biomass level

	**Experiment**	**Predicted: Gene expression**	**Predicted: Standard FBA**	**Predicted: Fitted FBA**	**Predicted: GIMME**	**Predicted: iMAT**
Ethanol	23.8	25.7	0	0	0	0
CO_2_	22.7	31.5	37.6	22.7	31.5	48.5
Glycerol	3.54	0	0	0	0	0
Acetate	0.311	0.016	0	0	0	0
Trehalose	0.0356	0.0301	0	0	0	0
Lactate	0.00873	0.0301	0	0	0	0
R^2^		0.87	-0.10	0.20	0.01	-0.71

**Table 2:** Comparison of experimental with predicted exometabolome fluxes, at 85% maximal biomass level

	**Experiment**	**Predicted: Gene expression**	**Predicted: Standard FBA**	**Predicted: Fitted FBA**	**Predicted: GIMME**	**Predicted: iMAT**
Ethanol	13.0	16.2	0	0	0	0
CO_2_	21.0	20.1	25.0	21.0	16.0	32.2
Glycerol	2.17	0.126	0	0	0	0
Acetate	0.239	0.00911	0	0	0	0
Trehalose	0.0215	0.0220	0	0	0	0
Lactate	0.00609	0.0176	0	0	0	0
R^2^		0.96	0.54	0.58	0.52	0.28

Fluxes are reported in units of mmoles/hr/g dry weight (DW), and are scaled by measured glucose uptake flux [see additional file – exometabolomics.xls].

Predicted fluxes are given for the gene expression constrained approach, introduced here, and the standard FBA method that relies upon maximisation of biomass. As standard FBA generates a number of feasible solutions, the “best” solution (the one which minimises the taxicab distance between prediction and data) is reported as “Fitted FBA”. Additionally, the existing algorithms GIMME [18] and iMAT [14] are applied, using the same model and gene expression data as was used to generate the gene expression constrained results.

It is recognised that the gene expression correlation method failed to predict glycerol transport at the 75% maximal biomass level, and underestimates this at the 85% maximal biomass level. Furthermore, acetate exchange is underestimated under both conditions. However, the gene expression method is capable of predicting low-level exometabolic fluxes of lactate and trehalose, and it is noticed that existing methods also investigated (GIMME [18] and iMAT [14]) did not predict anything other than carbon dioxide excretion when applied to the same model and gene expression data.

## Conclusions

In this paper, a methodology for using absolute quantitative gene expression data to predict genome-scale flux patterns has been defined. Unlike standard constraint-based analysis approaches such as biomass maximisation, this method is able to provide accurate predictions of the major exometabolome fluxes in *Saccharomyces cerevisiae* under the measured experimental conditions.

Both transcriptome and exometabolome data from steady state cultures grown under specific conditions of interest are generated. The acquisition of data from defined and controlled cultures allows the direct comparison of the two data sets. The relatively easy generation of data - commercial kits were used to extract the RNA from the cell - negates the need to use a “consensus” set of publicly available transcriptomics data. Moreover, with calibration it allows for absolute quantification, rather than the use of relative values as typically output in microarray experiments. Advancements in analytical technologies within the metabolomics field and the development of defined methodologies and tools have facilitated the generation of robust and reproducible data for systems biology studies.

The method described here does not rely on the definition, and assumed maximisation, of a biomass function that is often uncertain and necessarily depends on the specific growth conditions. It is notable that the proposed approach provides prediction of exometabolome fluxes under two growth conditions, whereas the standard FBA formulation provides a single prediction that is unable to distinguish between conditions. Tailoring the standard FBA approach to deal with different growth conditions would require the specification of individual biomass functions for each condition. This time-consuming task requires knowledge of the cell composition and energetic requirements under the growth condition of interest. The approach described here thus provides an easier means of determining metabolic fluxes under a range of conditions than relying on the standard method of biomass maximisation.

The gene expression constraining approach can also be considered as a filter: given that the experimental data can be taken as representative of the fluxes, the random errors associated with such data should cancel out at a genome-wide level. Because the FBA solution needs to satisfy the steady-state assumption, the predicted fluxes must be consistent with the mass conservation of each metabolite. If the topology of the network encompasses the whole system metabolism, the noise of the data should subside in favour of the real signal. Moreover it allows changes in ‘omics data from the perspective of their influence on the system as a whole to be viewed. Although transcriptomics data are not necessarily representative of the protein levels, their use in metabolic constraint-based modelling provides much better predictive power than does the standard FBA method. Specifically, the gene expression constrained method provides good fits to the measured flux to exit data at both 75% and 85% growth level *R*^2^ = 0.87 and *R*^2^ = 0.96 respectively), while the standard FBA method provides a much poorer fit.

Typically, gene knockouts are modelled under the assumption that there is no downstream effect on gene regulation, and that there is a minimum change at the flux level (for example using minimisation of metabolic adjustment (MoMA) [43]). Rather than simply making such an assumption, this work suggests a better approach would be to effect the (relatively easy) measurement of gene expression data after knockout, and subsequent use of this data to predict the new flux pattern. It is envisaged that the same approach could be used to retrieve flux patterns in cells under different physiological conditions, or during the progression of metabolic diseases such as cancer and diabetes. Furthermore, methods that predict exometabolomic fluxes are of particular use in ‘white’ biotechnology applications [44,45].

## Methods

### Continuous cultures of *Saccharomyces cerevisiae*

Steady-state cultures of *Saccharomyces cerevisiae* (BY4743 hoΔ/HO, (YDL227C; MATa/MATα; his3Δ1/his3Δ1; leu2Δ0/leu2Δ0; met15Δ0/MET15; LYS2/lys2Δ0; ura3Δ0/ura3Δ0) were established using a permittistat approach [46,47], in which the electrical capacitance of the culture was monitored by a probe inserted into the vessel connected to a Biomass Monitor 220 (Aber Instruments, UK). The cultures were grown in F1 media [48] with a working volume of 2l in a 3l vessel (Applikon Biotechnology, Netherlands). The bioreactors were operated at a temperature of 30°C, 650 rpm agitation control, pH 4.5 and 1.5 ml min^-1^ air supply, under the control of the operating software BioXpert (Applikon Biotechnology, Netherlands). A feedback loop was programmed to regulate the media pump and maintain the culture at the desired set point (either 75% or 85% of the maximum achievable biomass levels known for this strain in the growth media used). As usual in a turbidostat, the growth rate of the culture was at μ_max_ and without nutrient limitation. Off-gas analysis of O_2_ and CO_2_ was measured using a tandem gas analyser (Magellan Instruments, Sweden). Further details of the experimental procedure are outlined in [48].

Samples were collected for transcriptome and exometabolome analysis from three independent (biological) replicates operated under identical conditions. The growth parameters turbidity (OD680nm), dry weight, culture purity, extracellular glucose concentration and total cell count (determined with a Cellometer Auto M10, Nexcelem Biosciences, USA) were monitored to assess the reproducibility of the replicates.

### Generation and analysis of RNA-Seq data

The RNA was extracted from the *Saccharomyces cerevisiae* samples using a RiboPure yeast kit (Ambion Life Sciences, USA). Total RNA samples were depleted of abundant ribosomal RNA molecules using the RiboMinus eukaryote kit for RNA-Seq (Invitrogen, USA). The RNA transcripts were converted into a cDNA library using the SOLiD total RNA-Seq Kit (Applied Biosystems, USA). Analysis of the library was performed using a SOLiD sequencing system (Applied Biosystems, USA) for the whole transcriptome library, generating millions of short reads of sequence. Counts for each transcript were generated by first aligning sequence reads were aligned onto a yeast reference genome (SGD/sacCer2 assembly, downloaded 09/02/2011 from the UCSC Genome Browser [49]) using Bowtie, version 0.12.7 [50]. After alignment, the Cufflinks algorithm [51], version 1.02, was employed to infer the gene transcripts most likely present according to the aligned reads. Once assigned to their most likely source genes, Cufflinks was employed to calculate transcript abundance in terms of RPKM [52], an estimate of transcript counts normalised both locally (according to the length of the assigned transcript) and globally (the number of total mapped reads per million).

### Generation and analysis of exometabolome data

Exometabolome samples were collected by syringe filtration (0.2μm pore filter) of the culture to rapidly remove the biomass from the culture. The resulting filtrate was snap-frozen in liquid nitrogen for further analysis. The concentrations of acetate and ethanol were performed by enzymatic assays (Megazyme, Ireland). Gas chromatography mass spectrometry (GC-MS) quantified the remaining metabolites detected in the exometabolome. The chromatographic peak areas for the metabolites detected in the samples were compared to an externally constructed calibration curve. A stock solution containing glycerol, trehalose, glucose and fructose was prepared at a concentration of 10 mmol l^-1^. The stock solution was serially diluted to provide calibration standards in the range of 0.1-1000 μmol l^-1^. Aliquots of the calibration standards and exometabolome samples were lyophilised (HETO VR MAXI vacuum centrifuge attached to a RVT4104 cooling trap; Thermo Life Sciences, UK).

The lyophilised samples and standards were derivatised for GC-MS analysis. An aliquot (50μl 20 mgml^-1^O-methylhydroxylamine solution was added to each sample and heated at 40°C for 90 minutes. 50μl N-acetyl-N-(trimethylsilyl)-trifluoroacetamide (MSTFA) was added to the samples and heated at 40°C. Samples were analysed on an Agilent 6890N gas chromatograph and 7683 autosampler (Agilent Technologies, UK) coupled to a LECO Pegasus III electron impact time-of-flight mass spectrometer (LECO Corporation, USA). 1μl of the sample was rapidly introduced in to an injector operating at 250°C, with a split ratio of 1:10 and a helium flow rate of 0.8ml min^-1^ in constant flow mode. Metabolites were separated on a SPB50 phase column (30m x 0.25mm x 0.25μm; Supelco, UK) with a 50 min temperature gradient (initial temperature of 70°C was held for 4 min followed by a 5°C min^-1^ temperature ramp to 290°C which was held constant for 2 min). The chromatographic eluent was introduced to an electron impact mass spectrometer. The transfer line and ion source temperatures were 250°C. Mass spectral data was acquired in the range 70-600 Da with an acquisition rate of 10Hz. Processing of the raw data was performed with LECO ChromaTof v2.25 software to construct a data matrix (metabolite peak vs. sample number) including the chromatographic peak areas. Calibration curves were constructed for each metabolite to determine the concentration of the metabolites in the exometabolome samples.

Carbon balancing was achieved to 97% and 95% for the 75% and 85% maximum achievable biomass growth conditions, respectively [see Additional file 1 – exometabolomics.xls].

### Mapping gene expression data to metabolic reactions

The model, Yeast 5, is encoded and distributed in the Systems Biology Markup Language (SBML) [53]. Following the conventions implemented by many genome-scale metabolic models [54], many reactions in Yeast 5 are associated with genes and proteins via gene-protein-reaction (GPR) associations: Boolean statements connecting genes to reactions. For example, reaction r_0003 ((R,R)-butanediol dehydrogenase) is annotated with:

GENE_ASSOCIATION: YAL060W

Given this simple mapping from gene to reaction, the gene expression count of YAL060W = 0.0152 ± 0.00757 can be used to directly give a reaction weighting of r_0003 = 0.0152 ± 0.00757.

However, in general, GPR associations are not so simple, and there is a many-to-many mapping from gene to reaction. For example, reaction r_0250 (carbamoyl phosphate synthase) is annotated as:

GENE_ASSOCIATION: (YJR109C and YOR303W) or YJL130C

with associated gene expression data YJR109C = 0.156 ± 0.083, YOR303W = 0.0976 ± 0.033 and YJL130C = 0.126 ± 0.013. Consider first the Boolean AND relationship above; this means that the reaction is catalysed by a complex between the two gene products. Since the maximum complex concentration is given by the minimum concentration of its components, the weighting of the complex is defined as:

YJR109C:YOR303W = min(YJR109C,YOR303W) = 0.0976 ± 0.033

The OR relationship allows for alternative catalysts to each reaction; as such the total capacity is given by the sum of its components, so:

r_0250 = YJR109C:YOR303W + YJL130C = 0.224 ± 0.035

Note that the standard deviation above is estimated through the assumption that the variables are uncorrelated, allowing variances to be added.

### Algorithm implementation

All scripts required to map gene expression data to a metabolic model, and to generate flux predictions from the model are available as additional files. In addition, the metabolic model Yeast 5 and the experimental gene expression data used in this study are provided as additional files. The Cobra Toolbox for Matlab [55] is required to run the scripts. Results generated in this study used the Gurobi Optimizer 4.5.1 linear solver (Gurobi Optimization, USA).

## Competing interests

The authors declare that they have no competing interests.

## Authors' contributions

NS conceived the idea and led the project. CW performed the continuous culture. DL and CW generated the gene expression data. WD generated the exometabolome results. DL, KS and EM developed and implemented the algorithm. KS produced the results. KS and NS developed the Matlab scripts. All authors read and approved the final manuscript.

## Supplementary Material

Additional file 1 **Exometabolomics data.** Experimentally measured exometabolomic flux data, both unscaled and carbon-scaled. Data generated from cellular culture grown at both 75% and 85% maximum biomass level, in units of mmoles/hr/g dry weight. (XLS 37 kb) Click here for file

Additional file 2 **Yeast 5.**Genome-scale metabolic model of metabolism in Saccharomyces cerevisiae. Click here for file

Additional file 3 **Gene data 75%.**Gene expression data, generated by RNA-Seq, on cellular culture grown at 75% maximum biomass level, in units of reads per kilobase of transcript per million mapped reads (RPKM). Click here for file

Additional file 4 **Gene data 85%.**Gene expression data, generated by RNA-Seq, on cellular culture grown at 85% maximum biomass level, in units of RPKM. Click here for file

Additional file 5 **Results.**Matlab script that runs the analysis function, using the above model and gene expression data. Generates flux predictions and compares these to the above experimentally measured exometabolomic flux data. Click here for file

Additional file 6 **Analysis.**Matlab function that performs the method described in this work. Additionally provides implementations of the existing algorithms GIMME [18] and iMAT [14]. Click here for file

Additional file 7 **Gene expression mapper for enzymatic complexes (A and B).**Helper Matlab function that is used in mapping gene expression data to individual reactions. Called by analysis function. Click here for file

Additional file 8 **Gene expression mapper for isoenzymes (A or B).**Helper Matlab function that is used in mapping gene expression data to individual reactions. Called by analysis function. Click here for file
